# Population Digital Health: Continuous Health Monitoring and Profiling at Scale

**DOI:** 10.2196/60261

**Published:** 2024-11-20

**Authors:** Naser Hossein Motlagh, Agustin Zuniga, Ngoc Thi Nguyen, Huber Flores, Jiangtao Wang, Sasu Tarkoma, Mattia Prosperi, Sumi Helal, Petteri Nurmi

**Affiliations:** 1 Department of Computer Science, University of Helsinki, PL 64 (Gustaf Hällströmin katu 2), Helsinki, 00014, Finland, 358 451707064; 2 Institute of Computer Science, University of Tartu, Tartu, Estonia; 3 School of Computing, Engineering and Digital Technologies, Teessidde University, Middlesbrough, UK; 4 Department of Epidemiology, University of Florida, Gainesville, FL, United States; 5 Department of Computer Science and Engineering, University of Bologna, Bologna, Italy

**Keywords:** digital health, population health, modeling, health data, health monitoring, monitoring, wearable devices, wearables, machine learning, networking infrastructure, cost-effectiveness, device, sensor, PDH, equity

## Abstract

This paper introduces population digital health (PDH)—the use of digital health information sourced from health internet of things (IoT) and wearable devices for population health modeling—as an emerging research domain that offers an integrated approach for continuous monitoring and profiling of diseases and health conditions at multiple spatial resolutions. PDH combines health data sourced from health IoT devices, machine learning, and ubiquitous computing or networking infrastructure to increase the scale, coverage, equity, and cost-effectiveness of population health. This contrasts with the traditional population health approach, which relies on data from structured clinical records (eg, electronic health records) or health surveys. We present the overall PDH approach and highlight its key research challenges, provide solutions to key research challenges, and demonstrate the potential of PDH through three case studies that address (1) data inadequacy, (2) inaccuracy of the health IoT devices’ sensor measurements, and (3) the spatiotemporal sparsity in the available digital health information. Finally, we discuss the conditions, prerequisites, and barriers for adopting PDH drawing on from real-world examples from different geographic regions.

## Introduction

*Population health modeling*, the monitoring and profiling of spatially fine-grained prevalence of diseases and health conditions, is a critical and key aim for public health [1]. Having accurate and timely information about the citizens’ health is essential for informing health policy decision makers, for optimizing care delivery, and in general for improving health outcomes. Detailed profiling and monitoring of diseases can also guide response to emerging health threats such as pandemics, assist in care delivery logistical planning and resource allocation, and the early detection of localized health-related phenomena. A deeper understanding of diseases’ interrelationships and epidemiology is also foreseen to play a key role in the future of health care and in sustaining improved health outcomes [2].

Current solutions for monitoring and profiling diseases, such as curating and linking data from electronic health records (EHRs) and health surveys [3], are expensive and have limited spatiotemporal coverage and scale and mostly target developing medical conditions rather than offer insights that can be used to help design policies for their prevention or early detection. These limitations in the availability of information restrict the conclusions that can be drawn. As a result, current solutions are capable of estimating overall disease prevalence and identifying risk factors but unable to offer continuous insights into the current health of the citizens. Improving the scale and coverage of public health models, and consequently the insights about the health of the citizens, requires new ways to cost-effectively collect continuous information about disease onset, health of individuals, and factors affecting them.

**Figure 1.**
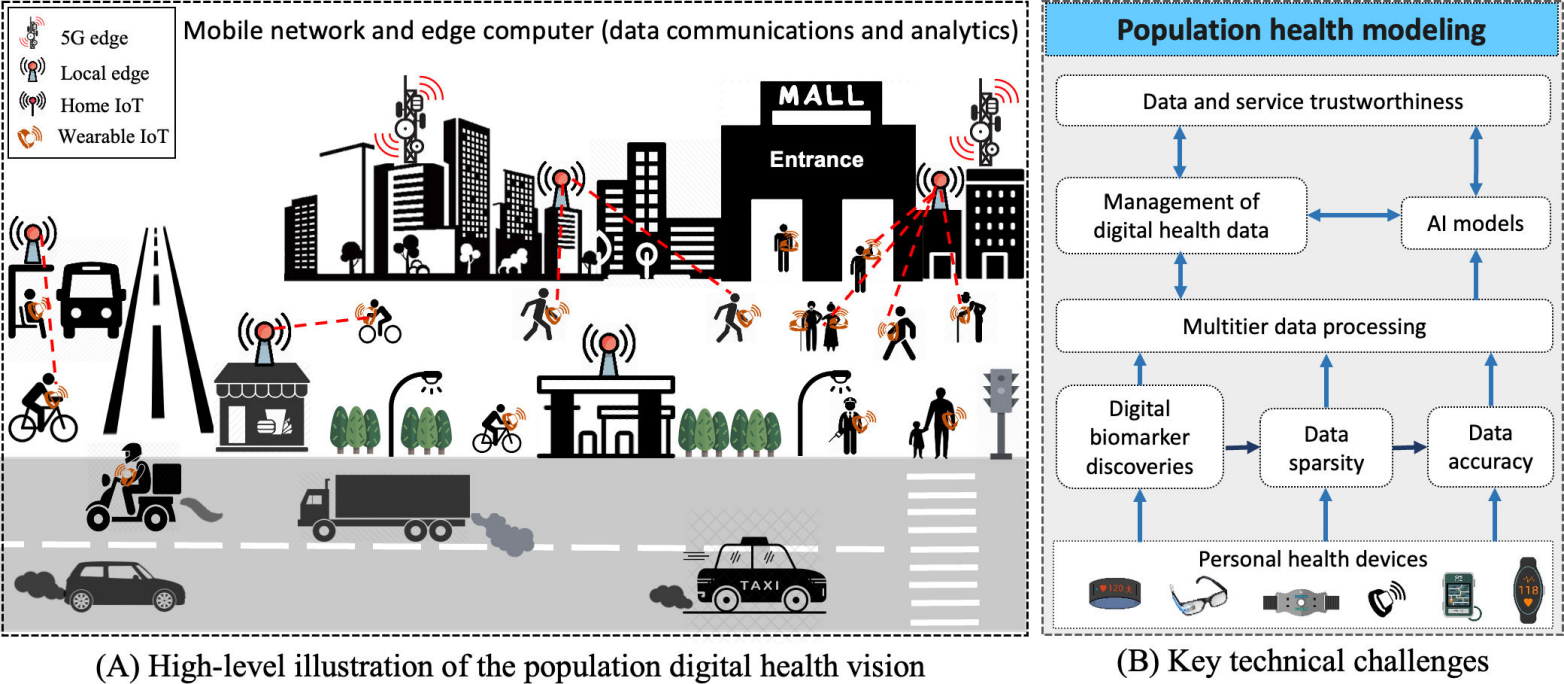
High-level overview of the potential of using personal health devices for population health modeling (A). The devices monitor individuals, and their data construct a continuous population health model that can be used as basis for health care and health policy decision-making. The key technical challenges that need to be solved are highlighted in the technical framework (B). AI: artificial intelligence; IoT: internet of things.

This paper introduces *population digital health* (PDH)—the use of digital health information sourced from health internet of things (IoT) and wearable devices for population health modeling—as an emerging research domain that offers an integrated approach for continuous monitoring and profiling of diseases and health conditions at multiple spatial resolutions. PDH is driven by the emergence and widespread adoption of digital personal technologies for health care, including health IoT devices and wearable technology for wellness and personal health monitoring, and advances in machine learning (ML) and artificial intelligence (AI) techniques capable of analyzing and extracting insights from complex real-world data streams by using powerful edge and cloud computing infrastructure that mobilizes intelligence and delivers requisite computational resources. Figure 1A and B show a high-level illustration of the PDH vision, highlighting the potential of using IoT and personal health devices as an alternative source of data that can modernize (digital) health monitoring, profiling, and reporting to support health care.

Realizing and adopting PDH modeling require addressing challenges in the way data are collected, analyzed, aggregated, and used to derive actionable insights, and lifting the barriers in technical and social hurdles relevant to the development of population health modeling (see section “PDH: A Research Agenda”). These challenges differentiate PDH and drive its research agenda. Specifically, PDH focuses on *population health*, that is, individual subcommunities or groups, instead of the general public, differentiating it from policy making, surveillance, and the modeling of health outcomes for the broader public, as explored by digital public health [4]. PDH targets etiology and identification of disease markers using wearables and other forms of digital data instead of targeting the diagnosis or treatment of diseases, as explored in (digital) precision medicine [5]. Similarly, while data from wearables and health IoT devices are used in mobile and pervasive health care [6-8], this differs from PDH which harnesses the data for modeling entire populations. Finally, while data are central tenet in many related fields, such as digital epidemiology [9] and precision public health [5,10], PDH specifically targets the challenges in enabling accurate and reliably continuous monitoring and profiling.

We present the overall PDH approach and highlight its key research challenges for PDH to establish a road map for delivering a highly accurate, cost-effective, scalable, equitable, and clinically trusted and actionable population health alternative. We also demonstrate the feasibility of PDH and highlight its benefit through three case studies targeting key challenges: (1) inadequacy of digital health information, (2) inaccuracy of sensor data on health IoT devices, and (3) spatiotemporal data sparsity in digital health information. The results demonstrate that PDH is a promising direction for increasing the scale and coverage of population health information and offers more detailed insights for modeling disease onsets, etiology, and other factors than what current population health modeling approaches can achieve. Eventually, we discuss the conditions and prerequisites that need to be satisfied to adopt PDH and draw examples of different geographic regions to highlight their current readiness.

## PDH: A Research Agenda

PDH brings together diverse sources of digital health information and uses it as input for population health modeling and for informing health policy making. Naturally integrating diverse information sources poses technological—and even societal—challenges that must be addressed. We next discuss key dimensions of PDH.

### Availability and Management of Digital Health Data

Personal health devices have long been envisioned as a powerful technology for supporting health monitoring [7], and the COVID-19 pandemic has further highlighted their potential as a mechanism to alleviate pressures on public health care delivery systems [11]. Relevant examples include blood pressure monitors, continuous glucose measurement devices, and smartwatches measuring physiological biomarkers, such as heart rate (HR), HR variability, blood oxygen saturation, and body temperature. What makes data from these devices particularly powerful is the diversity of available devices with powerful outreach to all population segments through smartphone apps and smart wearables on youth and work population and at-home medical monitoring devices [12]. At the same time, there are significant challenges in harnessing these data. First, EHRs have emerged over a long period of time and have well-structured representations, whereas currently available digital health information tends to follow proprietary formats and structures. This calls for data structures and algorithms that can consolidate data from different devices [13]. Second, digital health information is sensitive and may be stored and used over a long period of time, which requires combining privacy preservation techniques [14] with secure and tamper-free storage, for example, by taking advantage of distributed ledgers [15]. Finally, availability of digital health information is intrinsically linked to the use of such devices, which is governed by personal preferences, socioeconomic background of individuals, and other factors. As a result, the availability of specific types of information is biased toward certain population segments and there is a need to understand biases governing these divisions to ensure models developed from these data that are generalizable.

### Cooperation Between Private and Public Sectors

The integration of data from personal health devices into population health modeling necessitates the consent and cooperation of the companies producing these commercial products. Public-private partnerships (PPPs) present a promising avenue for achieving this as they offer an ethical and effective framework for integrating personal health device data into population health initiatives. These partnerships can be successful, however, only if there are standardized interfaces for integrating data from diverse personal health devices. In addition, the partnerships need to be based on binding and sufficiently long-term contracts to ensure the sustained availability of data from personal health devices. These contracts should outline clear guidelines for data sharing, privacy protection, and ethical data usage, providing a foundation for collaboration between commercial entities and public health systems. There is also a need for cost and profit-sharing models that incentivize the commercial sector to make data available for population health modeling, fostering a mutually beneficial framework for data sharing and utilization.

### Data Accuracy

Integrating data from personal devices with health care services requires accurate and reliable data that can be used to make sound policy decisions. Personal health devices, including devices for at-home use, are well known to be susceptible to errors unlike medical-grade devices and equipment [16,17]. Machine learning helps compensate these errors [18,19] and can even reach close to clinical accuracy in some situations [18] but significant challenges remain in ensuring their consistency (ie, robustness) and reliability in a wide range of everyday contexts. For example, in the context of HR monitoring, calibration techniques have been shown to be generally effective during regular physical activity, such as walking or biking but less so in contexts that feature activities with irregular motion patterns (eg, folding clothes). In addition, personal characteristics, such as how the device is used, worn, or the wearer’s skin complexion, can impact measurement accuracy [12,16,17]. Public health policies need to be based on accurate information and hence there is a need to understand potential errors and to have effective mechanisms to mitigate them. This requires replicable protocols for evaluating personal health devices for specific use cases. For example, studies on in-home monitoring of elderly people should be assessed with everyday activities they conduct at home as these can cause motion artifacts that distort the signal [20], whereas studies for using personal devices to screen heart conditions (eg, atrial fibrillation using a smartwatch) should be based on clinical criteria. While studies on understanding the performance of personal health devices are increasingly conducted, they tend to rely on different protocols, use different devices, have differing sample populations, and even reference devices [21]. Moreover, these studies are often anchored at clinical accuracy criteria rather than focusing on specific population health modeling needs, which makes it difficult to aggregate the devices into population health modeling processes. Indeed, showing a 5% error in HR estimates for a specific wearable device in walking and running does not provide sufficient insight into whether the device can be used for profiling or monitoring specific diseases. Replicable protocols anchored at specific population health targets can help make information more useful and easier to integrate.

### Regulations and Quality Standards

Ensuring the accuracy and reliability of personal health data is essential for informed decision-making in PDH. However, existing regulatory processes designed for clinical purposes, such as Food and Drug Administration regulations for medical devices, may not fully align with the characteristics and usage of personal health devices for population health purposes. Therefore, there is a need to update regulatory mechanisms to better accommodate personal health devices and to ensure their effectiveness and safe and acceptable use for population health. This may involve introducing more lightweight regulatory alternatives that are specifically tailored for collecting data for personal or population health purposes [22]. Beyond regulation, there is also a need to establish quality standards for data produced by personal health devices. Indeed, rigorous clinical standards, while essential, may not translate to the context of personal health devices due to the inherent variability in data collection procedures and usage contexts. This can be offset by deriving localized and contextualized quality standards that consider the specific contexts of use and the variability relative to the intended application, ultimately ensuring the reliability and validity of the data derived from personal health devices [23]. Failure to provide better regulations and to address the contextualization of data from personal health devices may lead to decreased user trust [24] and limit the potential to harness valuable data for population health.

### Digital Biomarker Discoveries

While some diseases and conditions such as cardiovascular diseases, diabetes, pulmonary diseases, and asthma have well-established digital biomarkers used in specialized personal health devices that can monitor their progression, the search is on for the most suitable digital biomarkers for many other diseases and conditions. Understanding the potential of a specific sensor or combinations of sensors and the information that can be gleaned from them in acting as representative digital biomarkers for certain diseases and conditions is currently shaping an exciting discovery pipeline. Such discoveries may exploit repurposing of sensors available in most smartphones, smartwatches, wearables, or at-home IoT devices [25]. For example, microphones from personal devices can sample audio clips to model the coughing sound of respiratory diseases [26]. In fact, speech sensing is currently being extensively researched as a promising source of digital biomarkers in multiple disease areas including Alzheimer disease, Parkinson disease, frontotemporal dementia, depression, and schizophrenia [27]. Motion sensors can also be used to detect early stages of Parkinson disease [28] or to analyze sleep patterns [8]. Research and advances in the digital biomarkers pipeline, through existing or novel sensors, are critically important to enabling PDH.

### Data and Service Trustworthiness

Digital population health requires that citizens trust the devices they use and how their data are being handled if they are to engage to guarantee that a critical mass of information is available. This can happen only if sensitive data are protected and there are no concerns about data misuse—a common concern in the use of health data [29].

Federated learning is seen as a potential way to aggregate EHRs [30] and could similarly be adopted for learning insights from health IoT and wearable devices as long as the accuracy of the data can be ensured. Yet, federated learning is vulnerable to poisoning where some of the data used to train the model or the model parameters are manipulated with the aim of misleading the model [31]. AI or ML algorithms are also vulnerable to model biases that may incorporate racial or socioeconomic differences [32] rather than capture the true causes of diseases. Population health and care delivery services will also require trustworthiness in the opposite direction if devices’ biomarker data are to be relied on and included within or alongside EHRs. This will require the use of verifiable digital identity for the users, for instance, using the emergent W3C Decentralized Identifiers concept [33] or implementing smart contracts between the concerned parties [34].

### AI Models and Data Sparsity

AI models are data-hungry, requiring vast amounts of data and labeled examples to operate effectively and accurately. Even at the population level, the available data tend to be sparse and hence there is a risk of the resulting models being unreliable.

Sparsity can also result in biases as the majority of the data tend to come from specific areas, times-of-day, or specific segments of the population. PDH modeling needs to be aware of these risks and have mechanisms to minimize their effects. For example, our previous work has shown that data reconstruction techniques can be effective at overcoming sparsity in EHRs [35,36] and similar techniques can be used on other forms of digital health data. Another issue related to AI modeling is the untapped opportunity of learning intra- and interdisease correlations brought by the diversity of the conditions and measurements contained in the collected data. Indeed, as outlined, health IoT technology monitors a range of different biometrics and there is a potential to combine and take advantage of such diverse data for constructing unprecedented, sophisticated PDH models.

### Data Biases

Bias is pervasive in health care data and has far-reaching implications for studies reliant on observational data, including those on population health modeling. Biases within EHRs often stem from socioeconomic or demographic disparities, such as studies being confined to specific age groups, genders, comorbidity-specific cohorts, or racially skewed populations [13]. The transition to data from personal health devices introduces further biases that are linked to technology and connectivity availability, cost-related barriers limiting access to specific devices or technology, and spatiotemporal biases arising from varying usage patterns. With PDH drawing on data from personal devices, there is a tangible risk of excluding certain socioeconomic groups from health studies. However, it should be noted that this issue also impacts EHRs as there are notable racial and socioeconomic disparities in the use and accessibility of health services. Addressing biases necessitates stringent reporting guidelines during the profiling and modeling phase to identify and rectify potential biases in modeling [37]. Robust techniques are also required to analyze and establish causal links between observations and population health outcomes [38]. Bias may also be mitigated by harnessing explainable AI techniques as they enable researchers to scrutinize how specific background variables may influence analysis outcomes [39].

### Multitier Data Processing

Collecting and analyzing digital health information produce vast amounts of measurements that need to be cleansed, aggregated, and preanalyzed. Low-level data may also need to be folded into digests to reduce data volume and facilitate better use. There is also a need to analyze certain measurements at different spatial and temporal resolutions to identify disease prevalence, for example, to identify risk factors in a specific district. However, the continuous transmission of data to remote servers—or the cloud—requires a lot from both the network and the remote infrastructure, besides posing privacy challenges and risks of unauthorized data access. This demands elasticity from the public health infrastructure to scale to increasing amounts of data volume and velocity. Such elasticity may become cost-prohibitive requiring intelligent use of edge computing [40]. Deploying AI support on the network edge can alleviate the burden and enable localized modeling that is tailored to specific geographic areas (eg, neighborhoods). Unfortunately, edge and fog solutions are neither scalable nor dense enough to provide continuous support for intermediate data processing. This requires multilayer architectures where each of the layers supports and participates in the processing. Advances in network connectivity, smart gateways, and cloud-fog-edge architectures make it possible to optimize and reduce the cost of moving and aggregating data, but this also requires carefully planned deployments. For example, deploying edge support at points-of-entry locations, such as malls, transportation stations, parks, or other similar locations that people frequent, can offer a cost-effective way to connect the majority of the population to the data processing infrastructure scalably (Figure 1). Developing suitable architectures and identifying practically feasible ways to deploy them are important challenges for ensuring large-scale feasibility of the PDH vision.

## Case Studies for PDH

### Availability of Digital Health Data

**Figure 2.**
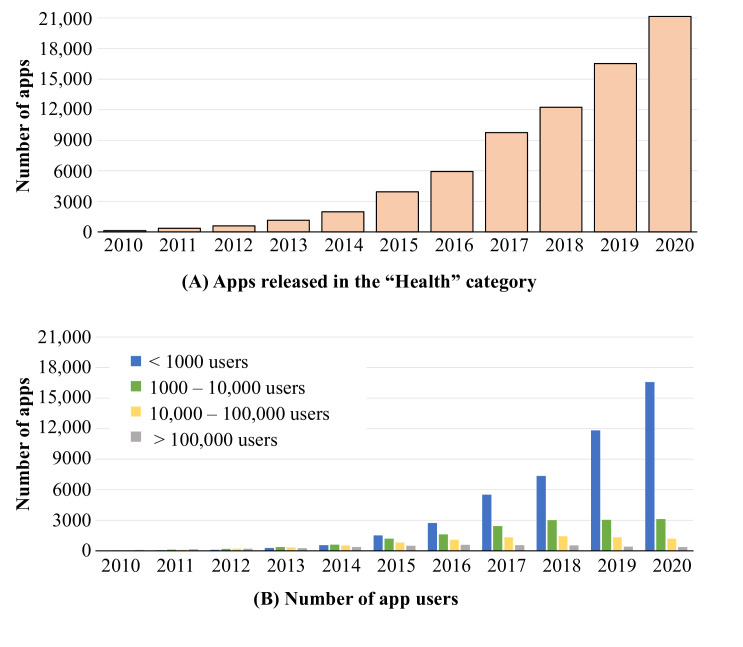
Number of smartphone apps released in the Health category between 2010 and 2020 (A) and number of app users (B).

Access to health data from health IoT devices and personal wearables is a prerequisite for PDH. We first show how data from health devices are increasing and can indeed significantly increase the scale and coverage of population health models. We use the Google Play Store Apps data set [41] and demonstrate how the number of apps and users per app have grown from 2010 to 2020. As shown in Figure 2A, the release of new applications has increased by an average of 66.9 (34.1%) per year.

At the same time, the adoption of apps has been very diverse, and the usage base tends to be highly fragmented. Indeed, the vast majority of health apps have fewer than 1000 users (39.1% [24.9%]), with only a small fraction of the apps (39.1% [24.9%]) having more than 100,000 users (Figure 2B). What this means in practice is that there are significant opportunities to take advantage of digital health data, but overall the user base tends to be fragmented and maximally taking advantage of all data can prove challenging. At the same time, a small number of apps garners a large user base and thus integrating data from them would serve as a logical starting point. This integration can harness either public application programming interfaces offered by the companies or, preferably, PPPs that set conditions and boundaries for data use. Beyond data fragmentation, there are naturally other challenges also in the use of the data. For example, all app ecosystems are prone to churn with a wide range of factors affecting the overall retention of apps [42].

The increased availability of digital health data alone is not sufficient as the data need to be suitable for modeling. Population health models commonly analyze records at a fixed spatial resolution (such as a postcode or a grid) but obtaining continuous measurements from personal health devices from all of the areas is next to impossible. We use the Carat [43] Top 1000 Users Long-Term App Usage Dataset [44] to highlight how data from health apps vary across time and depend on the app popularity. We focus specifically on the situation prior to the pandemic as this gives a more stable view of the app usage. Specifically, we analyze the daily collection patterns in 2017 and 2018 of the top three popular health apps used for tracking individual’s health in different contexts: (1) Samsung Health, (2) Fitbit, and (3) Sports Tracker Running Cycling.

**Figure 3.**
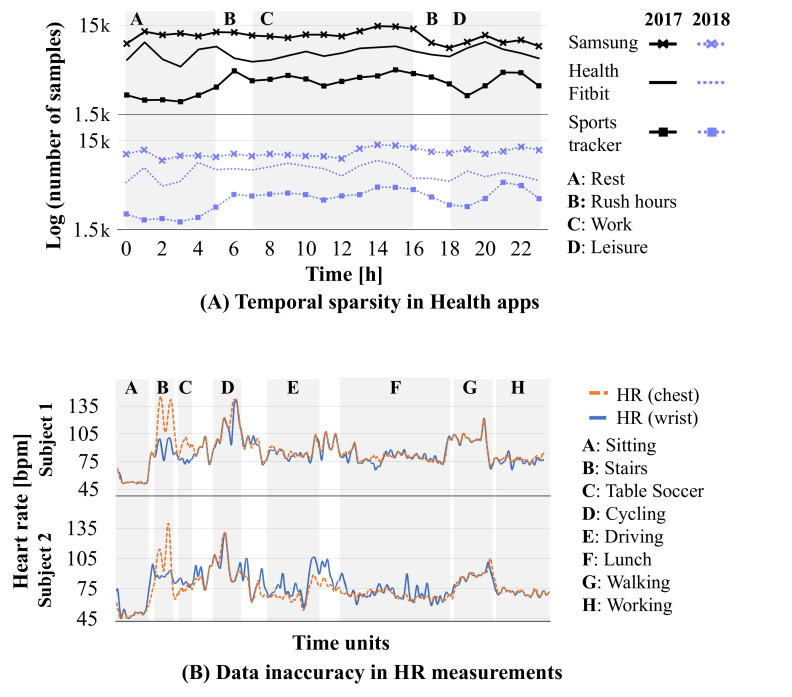
Temporal sparsity in Health apps (A). Data inaccuracy when measuring HR from different devices and body parts (B). HR: heart rate

Figure 3A shows that the usage patterns for the 3 apps generally follow diurnal patterns, which means that nights and mornings tend to have much lower amount of measurements than afternoons. While this tends to be a generic pattern for apps [45], naturally the usage patterns also vary depending on app functionality and other factors. For example, sleep-tracking apps naturally produce more data during nights than physical activity trackers. There are also some activity trackers that continuously collect measurements from different sensors and this is also the reason for the low variation in measurements for the Sports Tracker Running Cycling app. In these cases, most of the produced data do not contain any health-related data and hence there is a need for analyzing and validating which of the measurements are relevant for population health modeling purposes.

### Data Inaccuracy

We next demonstrate potential utilization of digital biomarker data and the effect that data sparsity and its accuracy have on modeling PDH. Personal wellness and health devices do not always meet clinical criteria for accuracy and thus the measurements need to be validated before they are used. We highlight this issue using HR measurements in the PPG-DaLiA data set [46]. The data contain HR measurements from a chest-worn device and a wrist-worn device to study HR variations during daily life activities [47]. Personal HR trackers are popular examples of devices producing digital health information and they are well known to be subject to inaccuracies [16].

Figure 3B shows the difference in HR measurements for the 2 devices (wrist-worn smartwatch and chest strap monitor) for 9 activities and 15 users. The HR variation is highest in aerobic activities (stairs: 104, SD 19.1 bpm, cycling: 112.4, SD 14.8 bpm, and walking: 93.8, SD 7.9 bpm) compared with activities with little movement (sitting: 53.1, SD 7.5 bpm, table soccer: 80.5, SD 8.6 bpm, driving: 78.7, SD 9 bpm, lunch: 75, SD 8.1 bpm, and working: 73.7, SD 4 bpm). The mean absolute error between the HR measurements collected at the chest and at the wrist is 7.7, SD 5.9 bpm, which is much worse than the reported accuracy of the devices and highlights issues with measurement quality. The discrepancy tends to be highest in activities where both the body and the wrist are moving (eg, stairs, 29.7, SD 13.9 bpm) and low motion activities result in lowest errors (eg, sitting, 2.6, SD 5.2 bpm). Integrating digital health information thus needs to be carried out carefully as otherwise errors in the measurements can result in misleading conclusions. Machine learning techniques can help curb such inaccuracies. Table 1 illustrates how even the simplest ML models can significantly decrease the errors by learning how to calibrate the sensors. The sole exception, in our example, is cycling where all algorithms slightly increase the error as they fail to capture the periodic nature of motion patterns. More complex algorithms, such as deep learning [19], can further reduce HR measurement errors, but they are similarly prone to overfitting on specific types of patterns. Further research is certainly needed to understand and mitigate different biomarker errors and to integrate this information reliably into public health models. At the same time, there is a need for regulations that specify what level of accuracy is needed, and these should be contextualized to consider how the data are being used. For example, using HR data to study the prevalence of obesity does not require the same accuracy as attempting to understand the prevalence of arrhythmia or other health conditions.

**Table 1.** Error (mean absolute error) of different heart rate calibration models.

Status	HR^a^ at wrist	Logistic regression	Random forest	Gradient boosting
Sitting	0.9 (0.9)	0.5 (0.8)	0.4 (0.7)	0.4 (0.5)
Stairs	34.9 (11.5)	10.2 (5.6)	12.7 (8.2)	10.4 (5.7)
Table soccer	17.9 (5.5)	4.2 (3.2)	3.3 (2.6)	3.0 (2.2)
Cycling	5.5 (9.6)	6.9 (6.2)	6.3 (8.1)	5.7 (6.5)
Driving	2.2 (1.9)	1.9 (1.5)	1.4 (1.2)	1.6 (1.2)
Lunch	2.5 (2.6)	2.4 (1.9)	1.9 (1.7)	1.9 (1.5)
Walking	0.7 (0.6)	0.7 (0.5)	0.5 (0.8)	0.7 (0.6)
Working	2.6 (1.9)	1.3 (1.3)	1.3 (1.2)	1.3 (1.1)
Overall	4.8 (9.2)	2.7 (3.6)	2.5 (4.3)	2.3 (3.4)

a HR: heart rate.

### Spatiotemporal Sparsity

**Figure 4.**
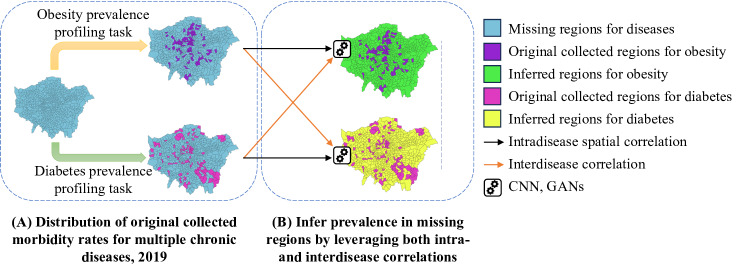
Recovery of missing prevalence data of 2 diseases from the London population health data set. Original prevalence data (A) contain many missing entries (blue areas in the left), which are augmented by exploiting spatial intradisease correlations (black arrows) and interdisease correlations between different diseases (orange arrows). This allows to obtain prevalence rate estimates for all geographic areas (B). CNN: convolutional neural network; GAN: generative adversarial network.

Finally, we show an application use case of how management and multitier data processing of digital health data can be used to reduce the sparsity of digital biomarker data and improve performance in PDH modeling. Our previous research has addressed spatiotemporal data sparsity in EHRs and developed a methodology that is based on deep learning and data reconstruction to mitigate the effects of sparsity [35,36]. The approach, coined *compressive population health* (CPH), uses intra- and interdisease correlations, convolutional neural networks, and generative adversarial networks to infer (recover) missing prevalence rate entries of different chronic diseases from a sparse population health data set (Figure 4).

Through experiments carried out on a decade of public health data containing 17 chronic diseases and health conditions across 500+ wards in London (the London population health data set [48]), research has shown that CPH is highly effective in modeling disease prevalence. The 2-stage reconstruction and fusion framework of CPH outperformed all baselines and achieved significantly improved accuracy on estimating prevalence rates. The extent of improvements, however, depends on the specific disease or health condition. For example, for obesity, CPH results in an error of 10.5%, an 8.5% improvement over the best baseline. For hypertension, CPH error is 2.7% but the baseline reconstruction techniques also perform well and the CPH improvement is only 5.1%. For diabetes, CPH achieves an error of 8.2%, outperforming the best baseline by 16.9%. In terms of coverage, sampling just 11% of the entire region can result in a lower than 15% reconstruction error for the missing data entries, suggesting that reconstruction can also improve the accuracy of the data. In contrast, other baseline methods need to sample at least 57% of the region to satisfy the same requirement. Overall, the results show that CPH can save more than 90% of resources in data collection while increasing the quality of data and the accuracy of estimates derived from it. Surely, further work is needed to address other factors beyond prevalence. Nevertheless, these results demonstrate the potential digital population health can have on significantly cutting cost of monitoring while improving coverage (and hence health equity) and data accuracy. From an analytics standpoint, CPH offers increased flexibility compared with traditional spatial epidemiology modeling, which is often limited to parametric-linear approaches and bound to low-dimensional measurement sets.

## Adopting PDH

The adoption of PDH is contingent not only on the identified challenges but also on the presence of a comprehensive ecosystem and network to support its implementation. The readiness of different cities, countries, or regions to fully embrace PDH varies significantly and is influenced by factors such as public willingness to share data, the availability of private-public partnerships, trust in the system, the existence of legal frameworks for health data, technological foundations, and the availability of health care providers and institutions to benefit from digital population health.

Regions with established clinical research networks exemplify ecosystems that can readily adopt digital population health, as they possess the necessary legislative frameworks, data and computing frameworks, and connections between stakeholders. An illustrative example is the OneFlorida+ Clinical Research Network [49], which integrates a data trust that offers access to curated EHRs, vital statistics, and Medicaid and Medicare claims. The data representation follows a common model, specifically the PCORnet Common Data Model [50], and adheres to Health Insurance Portability and Accountability Act regulations on health data privacy [51] providing interoperability and legislative protection on privacy. The network has been successfully leveraged to profile and analyze the prevalence of health conditions and diseases in the state of Florida, with examples including studies on hypertension [52] and adult obesity [53].

Another example is the shared European Health Data Space initiative across EU member countries that links curated health data records across EU member countries and aligns the data representation with data governance frameworks such as the General Data Protection Regulation and the EU Data Act [54]. These examples illustrate that regions with established networks for health data usage generally offer a strong starting point for adopting PDH, as they ensure the necessary infrastructure for curating, storing, and representing that the data are available, and that this infrastructure links with health care providers, patients, clinicians, and researchers, while being supported by robust legal frameworks. Smaller-scale examples include Estonia, which has strong data protection laws, widespread public trust in digital services, and a well-developed e-governance infrastructure [55], and Singapore, which has fostered PPPs in the health care sector and focused on creating a robust computing infrastructure [56].

While existing ecosystems provide a strong starting point for adopting PDH, adoption is also possible without such networks, provided that a sufficiently large percentage of the population uses personal health devices and companies consent to their data being used for health purposes, or that suitable PPPs are established. Many developed countries fall into this category, as they have widespread adoption of personal health devices but limited access to health services, let alone having unified data models and data governance models. Thus, the adoption of PDH is not restricted to a specific model or framework, but different models can be followed depending on the structure of the regional health care service networks.

While there are many possibilities to adopt PDH, there are also negative scenarios where adoption may be hampered. First, maintaining a sufficient level of trust among individuals to share their data is crucial, and misuse of personal data can erode this trust. Breaches of health care data have become increasing common, which is degrading the user’s willingness to share their personal health data [57]. Similarly, inadequate standards for representing digital health data and the evolving nature of digital health technology pose challenges in integrating data from different providers. Many regions still have inadequate standards for representing digital health data and this can make it hard to integrate data from different provides [23,58]. Regions with existing standards are better positioned to harness digital data, but at the same time digital health technology continues to evolve and new devices and health indicators emerge regularly. Thus, even if standards exist, they need to be updated frequently as new tools and technologies are developed. Regulations and legislative frameworks, while essential for ensuring data safety, also create barriers [59]. Finally, inequity of health access is another concern that can hamper adoption, as certain population segments have unequal opportunities to access digital health tools and technologies. For example, older people and those from lower income brackets tend to use these technologies fewer than other parts of the population [60]. Thus, achieving equitable reach across all population segments may require PDH to coexist with another approach that reaches those segments that personal health devices fail to reach.

Despite these challenges, the increasing use of digital health technologies and the evolving societal attitudes toward their adoption indicate a growing receptiveness to PDH. As long as significant breaches of sensitive health data are avoided, the trend toward adoption is likely to continue, highlighting the inevitability of society becoming increasingly amenable to embracing PDH.

## Summary and Conclusion

We presented PDH as an emerging research domain that harnesses digital information provided by wearables and health IoT devices for population health modeling. We highlighted key research challenges for PDH, relating to the availability, readiness, and management of health data; the inaccuracy inherent in these data and the spatiotemporal sparsity of the data measurements; and the trustworthiness of the overall ecosystem. PDH complements existing population health modeling approaches by increasing the scale, coverage, and power of the models to explain onset, causation, and other factors about diseases and health conditions. Through case studies, we demonstrated how PDH can indeed increase the scale and accuracy of population health models. We also demonstrated how ML and AI are essential for tackling issues in data quality. Finally, we discussed the necessary conditions for transitioning to PDH and how different regions can adopt it. Our research takes the first steps toward establishing the viability of a new approach for public health modeling and demonstrating the role machine intelligence plays in it.

## Abbreviations

CPH: compressive population health
EHR: electronic health record
IoT: internet of things
ML: machine learning
PDH: population digital health
PPP: public-private partnership
